# Non-Intrusive Early Insulation Fault Detection for Induction Motors Using a Dual-Frequency Microstrip Antenna Array Based on UHF Partial Discharge Electromagnetic Wave Detection

**DOI:** 10.3390/s26103126

**Published:** 2026-05-15

**Authors:** Yinghua Xu, Yongfeng Wu

**Affiliations:** School of Electrical and Information Engineering, Hunan Institute of Engineering, Xiangtan 411104, China; 70211@hnie.edu.cn

**Keywords:** induction motor, early insulation fault, non-intrusive detection, microstrip antenna array, fault detection

## Abstract

Aiming at the problems that existing detection methods struggle to accurately identify early insulation faults of induction motors, are susceptible to interference, and have poor installation adaptability, a non-intrusive detection method for early insulation faults of induction motors based on a microstrip antenna array is proposed. Relying on the low-loss electromagnetic wave transmission characteristic of the heat dissipation hole at the tail of the induction motor, a four-element microstrip antenna array with multiple narrow beams and dual detection frequencies is designed, with the detection frequencies accurately set at 1.14 GHz and 2.23 GHz, which effectively avoids the motor operation noise frequency band (≤300 MHz) and the strong interference frequency band of mobile base stations (900 MHz, 1.8 GHz, 2.4 GHz). Utilizing the high gain and strong directivity of the array antenna, the accurate extraction and amplification of weak electromagnetic wave signals from early insulation fault discharge penetrating through the heat dissipation hole are realized. The full-dimensional simulation design of the antenna array is completed by using HFSS electromagnetic simulation software, and an industrial-grade experimental platform is built to carry out multi-condition verification experiments. The results show that the proposed detection system can realize non-intrusive, non-stop, and non-disassembly identification of early insulation discharge faults in induction motors, with a fault recognition rate of 94% for single faults and 90% for composite faults, and the average signal-to-noise ratio reaches 31.6–35.2 dB. Even under strong industrial electromagnetic interference, the recognition rate remains above 85%. This method overcomes the problems of traditional methods such as severe noise interference, difficult installation, and inability to monitor online, providing a high-efficiency scheme for real-time insulation state monitoring of industrial induction motors with good engineering application value.

## 1. Introduction

As the core power equipment in industrial production, induction motors are widely used in key industries such as chemical engineering, metallurgy, mining, and machinery manufacturing, consuming more than 70% of total industrial power. Their operational reliability directly determines the safety, stability, and production efficiency of industrial systems. Induction motor faults are mainly categorized into mechanical faults and electrical faults, among which electrical insulation faults are the primary cause of unplanned shutdowns. During fault evolution, mechanical faults often induce secondary electrical failures such as insulation damage and short-circuit breakdown. Therefore, the actual service life of an induction motor is essentially determined by its electrical insulation life [1,2,3,4,5]. Early insulation faults are mainly characterized by partial discharge and weak spark discharge, featuring low amplitude, inconspicuous characteristics, and poor identifiability. Without timely and accurate detection and early warning, such faults will gradually develop into serious failures including turn-to-turn short circuit and phase-to-phase short circuit, which not only damage equipment but also cause systemic production losses and even safety accidents.

At present, the mainstream fault detection methods for induction motors mainly include electrical parameter analysis, pulse current method, temperature analysis, and vibration signal analysis. Electrical parameter analysis extracts fault features from voltage and current waveforms, but waveforms are easily distorted by load variations, and weak discharge signals are often submerged by strong power signals, making effective extraction difficult. Reference [6] proposed a monitoring method based on diagonal elements of the negative-sequence impedance matrix, which can eliminate errors from load, operating conditions, and measurement, but requires off-line motor data and additional equipment. Reference [7] proposed a method to detect turn faults by injecting high-frequency negative-sequence voltage components, providing a new diagnostic idea for inverter-fed or soft-start motors. Although it affects normal operation to a certain extent, the impact is limited due to the short injection time. Reference [8] pointed out that when a turn-to-turn short circuit occurs, the envelope of the stator’s three-phase current fluctuates at the power frequency cycle, and the second harmonic in the steady state can be used as a characteristic for turn fault analysis. Reference [9] realized single-turn winding short-circuit monitoring by analyzing the second harmonic of q-axis current, but it was still limited to steady-state analysis. Reference [10] adopted neural network algorithms to extract fault features, which can effectively suppress noise and unbalanced power supply after sufficient training. To improve model performance, reference [11] further adopted feedforward neural networks and fuzzy neural networks; however, these methods suffer from insufficient real training data, mostly relying on simulation samples, resulting in weak generalization and limited to specific motors. The pulse current method collects partial discharge pulse currents generated by insulation defects via coupling capacitors. Reference [12] conducted multi-factor aging tests on stator windings and concluded that using only the maximum discharge magnitude to evaluate insulation aging status is inaccurate. References [13,14] studied the partial discharge characteristics of 10 kV stator windings under corona, slot discharge, and other defects, and proposed establishing a partial discharge fingerprint database. Reference [15] analyzed the mechanism and fingerprint patterns of phase-to-phase discharge in 10 kV three-phase induction motors. However, all these methods require installing coupling capacitors or high-frequency current sensors at winding terminals or neutral points, posing wiring safety risks and interference introduction. The method is sensitive to inverter carrier signals, switching operations, adjacent equipment corona, and ground loop currents, leading to misjudgment in strong electromagnetic environments and requiring strict shielding and filtering. Temperature analysis includes sensor-embedded and sensorless schemes. Resistance temperature detectors and thermocouples are commonly embedded in stator windings. Reference [16] proposed a temperature monitoring method suitable for soft-start motors by injecting a short-time DC bias voltage to measure winding resistance, with negligible impact on system performance. Reference [17] monitored motor cooling using a first-order thermal model, but accurate results require stable working conditions. Reference [18] improved accuracy using an adaptive Kalman filter. Reference [19] proposed a novel transfer-function-based thermal model with fewer parameters, comprehensive loss consideration, and error controlled within 4 °C. Vibration signal analysis identifies mechanical faults such as bearing and shaft failures by extracting vibration features, but weak vibration from early insulation discharge is easily submerged by operating noise and difficult to identify effectively. Reference [20] proposed rolling bearing fault diagnosis based on the fusion of vibration and current signals. Reference [21] realized bearing fault recognition through wavelet decomposition and neural networks. Reference [22] established a basic bearing fault model using spectrum analysis. Reference [23] performed wavelet analysis on Park vector current signals and used RMS values of subband coefficients as eigenvalues for bearing fault diagnosis. Reference [24] improved the traditional LMS adaptive filter by combining wavelet transform, continuous zoom Fourier transform, and enhanced LMS algorithm for bearing fault detection of induction motors. All the above methods require power outage and disassembly for sensor installation, resulting in extremely poor adaptability to operating equipment, and cannot meet the actual demand of online insulation condition monitoring for induction motors in industrial sites.

To overcome the shortcomings of existing technologies, this paper proposes a non-intrusive early insulation fault detection method for induction motors based on electromagnetic wave detection. Using the characteristic that electromagnetic waves in 300 MHz–3 GHz generated by insulation discharge can penetrate the motor’s tail cooling holes with low loss, a dedicated microstrip antenna array is designed to realize directional and high-sensitivity reception of fault electromagnetic signals. This paper systematically expounds the detection principle, completes the simulation design and hardware fabrication of the antenna array, and comprehensively verifies the feasibility, detection sensitivity, anti-interference performance, and engineering adaptability of the method through prototype development, experimental platform construction, multi-condition fault simulation, and anti-interference tests. The results provide a solid theoretical and experimental foundation for the engineering application of this technology.

## 2. Detection Principle

### 2.1. Analysis of Electromagnetic Wave Characteristics of Induction Motor in Operation

**Figure 1:**
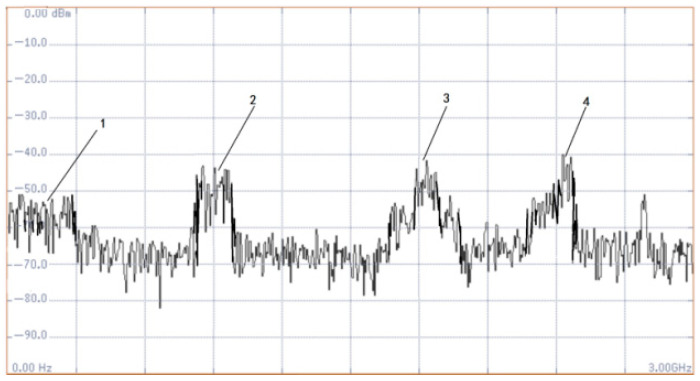
Background noise spectrum of induction motor in operation.

To clarify the electromagnetic wave frequency characteristics of induction motors in operation and fault states, the electromagnetic wave frequency measurement is carried out on induction motors of different brands and models. The results show that during the normal operation of the motor, the frequency of the electromagnetic field generated by the electromagnetic induction of the stator and rotor, mechanical rotation, etc., is all ≤300 MHz, which is the background noise frequency band in the detection process; at the same time, there are strong interference frequency bands of mobile base stations such as 900 MHz, 1.8 GHz and 2.4 GHz in industrial sites, as shown in Figure 1. Such frequency bands have high signal strength and are prone to superposition interference on fault detection signals, affecting detection accuracy.

However, the partial discharge and spark discharge generated by insulation defects inside the induction motor will radiate electromagnetic waves in the wide frequency band of 300 MHz~3 GHz due to the breakdown of insulation medium and rapid charge release. This frequency band is not only far from the noise frequency band of normal motor operation, but can also effectively avoid the strong interference frequency band of base stations through reasonable design of detection frequencies, providing a good frequency domain basis for the accurate extraction of fault characteristic signals.

Induction motors are packaged with thick metal casings with extremely small gaps on the body, and the electromagnetic wave penetration loss is >80 dB, making it difficult for fault-generated electromagnetic waves to effectively penetrate the metal casing for outward transmission; however, to ensure motor heat dissipation, each motor is provided with a circular heat dissipation hole (diameter 10~20 cm) at the tail, which is the only effective channel for the outward transmission of electromagnetic waves inside the motor. The measured electromagnetic wave penetration loss of this channel is <10 dB, providing an important physical basis for the non-intrusive detection of early insulation faults of induction motors.

### 2.2. Detection Theory of Array Antenna

To achieve high-sensitivity reception of weak discharge electromagnetic wave signals, this method adopts an array antenna to replace the traditional omnidirectional antenna and utilizes the array gain effect to improve the signal receiving power, effective detection distance and detection sensitivity. In free space, the calculation formula of antenna receiving power is

(1) $$P_{r}=\frac{P_{t}G_{t}G_{r}\lambda^{2}}{(4\pi R{)}^{2}L}$$

where Pr is the receiving power, Pt is the transmitting power, Gt is the transmitting antenna gain, Gr is the receiving antenna gain, λ is the operating wavelength, R is the linear distance between the transmitting and receiving antennas, L is the additional system loss, and 4π is the solid angle coefficient of free-space radiation.

Under ideal conditions, the gain of the omnidirectional antenna Gr = 1, and the maximum gain of the N-element array antenna is approximately Garray ≈ N. Under the same transmitting power and operating frequency, the following holds:

The receiving power of the omnidirectional antenna is

(2) $$P_{r,\mathrm{iso}}=\frac{P_{t}G_{t}1\lambda^{2}}{(4\pi R{)}^{2}L}$$

The receiving power of the array antenna is

(3) $$P_{r,\mathrm{array}}=\frac{P_{t}G_{t}G_{\mathrm{array}}\lambda^{2}}{(4\pi R{)}^{2}L}$$

The ratio of the receiving power of the array antenna to that of the omnidirectional antenna is

(4) $$\frac{P_{r,\mathrm{array}}}{P_{r,\mathrm{iso}}}={\;G}_{\mathrm{array}}=N$$

The corresponding gain and sensitivity improvements are

(5) $$G_{\mathrm{array},\mathrm{dBi}\;}=10\mathrm{lgN}$$

(6) $$\Delta S={\;G}_{\mathrm{array},\mathrm{dBi}}$$

The improvement multiple of the equivalent communication distance of the array antenna relative to the omnidirectional antenna is

(7) $$\frac{R_{\mathrm{array}}}{R_{\mathrm{iso}}}=\sqrt{G_{\mathrm{array}}}$$

### 2.3. Design of Array Antenna

Based on the above array antenna detection theory and the measured analysis of the physical size of the heat dissipation hole of the induction motor, the performance comparison results of array antennas with different element numbers and omnidirectional antennas are obtained, as shown in Table 1. It can be seen from Table 1 that the higher the number of array elements, the more significant the improvement effect of the receiving power, detection distance, and sensitivity of the array antenna. However, in the detection frequency range of 300 MHz~3 GHz, the size of the antenna elements is fixed. If the number of elements is too large, the overall size of the antenna array will exceed the heat dissipation hole of the induction motor (the diameter of the heat dissipation hole of motors below 10 kV is ≤20 cm), and some beams will deviate from the detection area, which will instead reduce the detection sensitivity. Considering the detection performance and installation adaptability comprehensively, the four-element scheme is determined as the optimal design, which can simultaneously take into account the array gain of 6.0 dBi, four times the receiving power improvement and the compact size requirement, and is fully adapted to the installation space of the heat dissipation hole at the tail of the induction motor.

The core design objectives of the microstrip antenna array are: to effectively avoid noise and base station interference frequency bands, to achieve full-coverage detection of the heat dissipation hole through multiple narrow beams, and to realize high-gain directional reception of fault signals. Based on this objective, the designed four-element microstrip antenna array includes two high-frequency elements (receiving frequency band 2.1~2.4 GHz) and two low-frequency elements (receiving frequency band 1.0~1.5 GHz). The performance optimization of the antenna array is realized by optimizing the element size and element spacing.

#### 2.3.1. Element Size Optimization

The element area directly affects the number of receiving frequencies and gain of the antenna. The larger the element area, the lower the optimal receiving frequency; the element spacing affects the number of beams and gain. Through the single-factor variable experiment, the element width is fixed at 37.2 mm, the element height is changed, and the receiving frequencies and gains of the antenna at different heights are tested, with the results shown in Table 2 and Table 3.

The experimental results show that when the height of the high-frequency element is greater than or less than 28.1 mm and the height of the low-frequency element is greater than or less than 60 mm, the antenna will have multiple receiving frequencies, and most of the frequencies are in the noise or base station interference frequency bands, which are prone to interference during detection. Therefore, the optimal size of the high-frequency element is determined to be 28.1 × 37.2 mm, which can realize single-frequency reception at 2.23 GHz with a gain of 15 dB; the optimal size of the low-frequency element is 60 × 37.2 mm, which can realize single-frequency reception at 1.14 GHz with a gain of 16 dB, effectively avoiding multi-frequency interference and ensuring the purity of signal reception.

#### 2.3.2. Element Spacing Optimization

Fixing the above optimal element sizes, the element spacing is changed, and the influence of different spacings on the number of beams and gain is tested, with the results shown in Table 4 and Table 5. The experimental results show that when the distance between high-frequency and low-frequency elements and the distance between high-frequency elements are changed, the gains of the left, middle, and right three beams of the antenna will be unbalanced, and the gain of some beams is extremely low, which easily causes detection blind areas; when all element spacings are set to 23 mm, the intensities of the three beams reach consistency, with the gain distributed between 5.3 and 5.9 dB and no blind area caused by weak beams.

**Figure 2:**
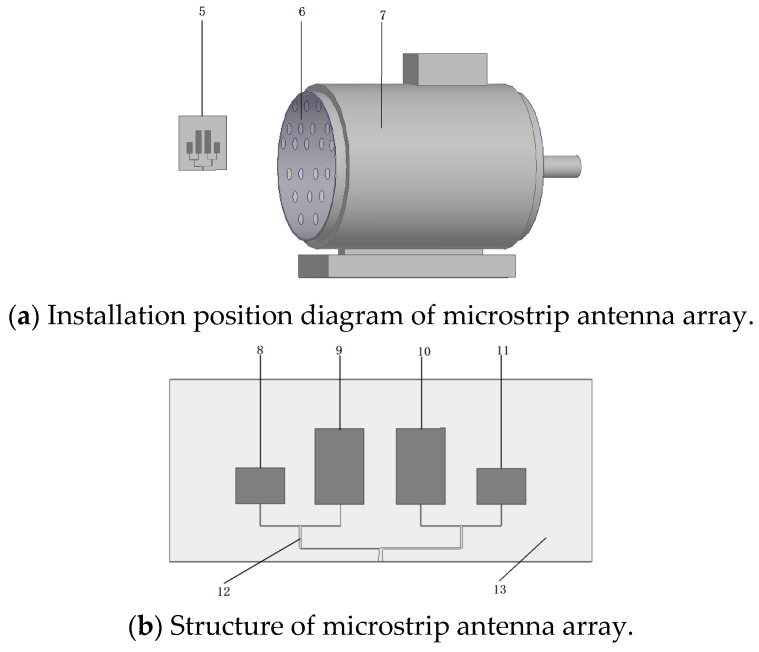
Structure and installation diagram of microstrip antenna array.

Based on the above research, a microstrip antenna array with multiple narrow beams and multiple detection frequencies is finally designed, which is installed at the rear of the heat dissipation hole of the induction motor, as shown in Figure 2a. The label 7 is the induction motor body, 6 is the heat dissipation hole, and 5 is the microstrip antenna array. The receiving frequencies of the microstrip antenna array are designed at 1.14 GHz and 2.23 GHz, which can avoid the motor noise frequency band and the mobile base station signal frequency band. When early insulation discharge occurs inside the motor, the generated electromagnetic wave signals will be released through the heat dissipation hole. Since the designed microstrip antenna array has multiple narrow beam gains and all its directions point to the heat dissipation hole, it has extremely high detection sensitivity to the inside of the heat dissipation hole and can accurately detect the weak partial discharge electromagnetic wave signals penetrating through the heat dissipation hole.

A microstrip antenna array based on a PCB board is designed according to the above detection principle, as shown in Figure 2b. The PCB dielectric material 13 adopted by the microstrip antenna array is FR4 with a dielectric constant of 4.4 and a thickness of 1.6 mm. There are four electromagnetic wave-receiving elements on the PCB dielectric material 13. Elements 8 and 11 are high-frequency elements with a size of 28.1 × 37.2 mm, used for receiving electromagnetic wave signals in the 2.23 GHz frequency band; elements 9 and 10 are low-frequency elements with a size of 60 × 37.2 mm, used for receiving electromagnetic wave signals in the 1.14 GHz frequency band, and the spacing between elements is 23 mm. The electromagnetic wave signals received by these four elements enter the receiver through impedance matching network 12 for amplification and detection.

## 3. Modeling and Simulation

The HFSS electromagnetic simulation software is used to carry out full-dimensional simulation analysis of the four-element microstrip antenna array, focusing on verifying the return loss, impedance matching, electromagnetic field distribution, and radiation direction characteristics of the antenna. At the same time, the electromagnetic wave transmission characteristics when early insulation faults occur inside the induction motor are simulated, providing a solid theoretical basis for subsequent experimental tests.

### 3.1. Performance Simulation of Microstrip Antenna Array

#### 3.1.1. Return Loss Characteristics

**Figure 3:**
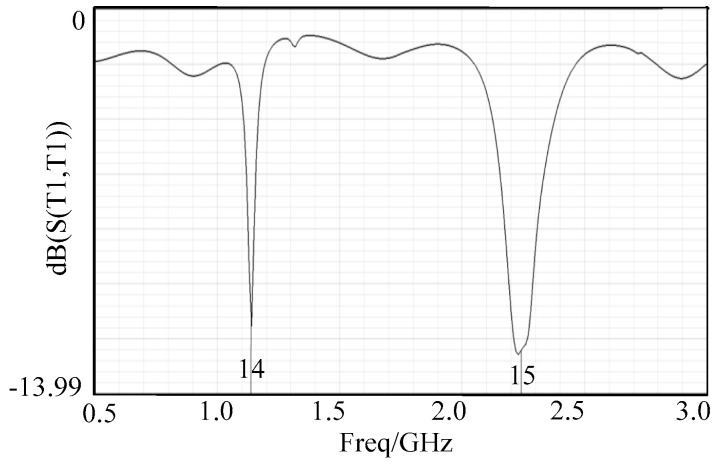
Return loss of microstrip antenna array.

Return loss is an important index for measuring the antenna’s signal receiving efficiency. The lower the return loss value, the higher the receiving efficiency of the antenna for the target frequency band signal and the smaller the signal reflection loss. The simulation results show that the designed antenna array has obvious loss valleys at the two target detection frequencies of 1.14 GHz and 2.23 GHz, as shown in Figure 3, with return losses of −11.5 dB and −28.1 dB respectively, both far lower than the industrial qualified threshold of −12.6 dB, indicating that the antenna has extremely high receiving efficiency for electromagnetic wave signals at the target detection frequencies and no obvious signal reflection loss, and can effectively receive fault-generated electromagnetic wave signals.

#### 3.1.2. Impedance Matching Characteristics

**Figure 4:**
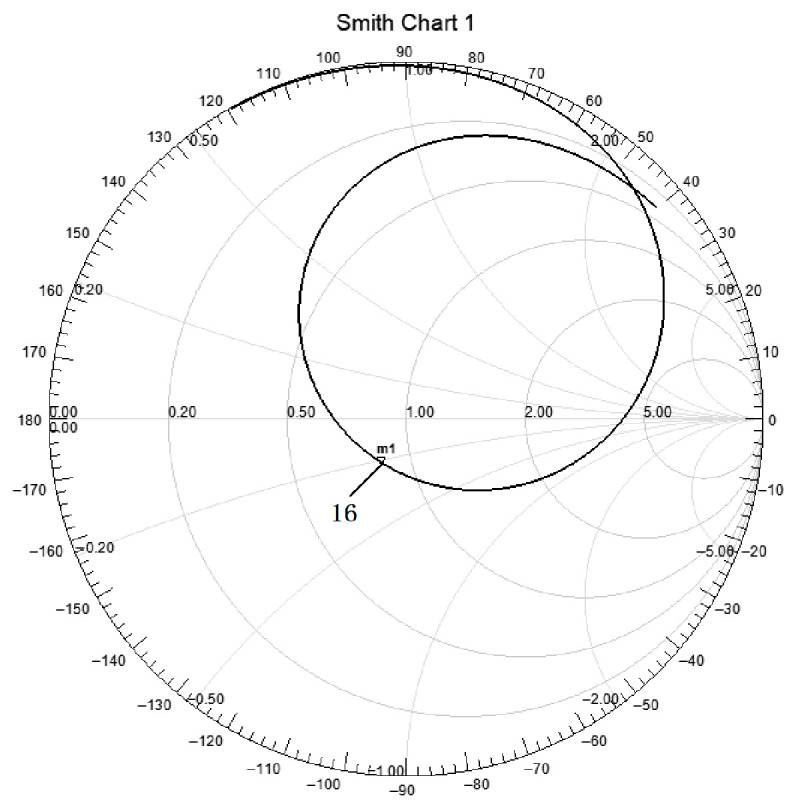
Smith chart of microstrip antenna array.

The Smith chart simulation results show that, as shown in Figure 4, the impedance points of the antenna array at the detection frequencies of 1.14 GHz and 2.23 GHz are both close to the center of the Smith chart, achieving good impedance matching; through the design of a special impedance matching network, the antenna output impedance can be stably maintained at the standard value of 50 Ω, ensuring the seamless connection between the antenna and the radio frequency receiver, minimizing the loss during signal transmission, and ensuring the integrity of signal transmission.

#### 3.1.3. Electromagnetic Field Distribution Characteristics

**Figure 5:**
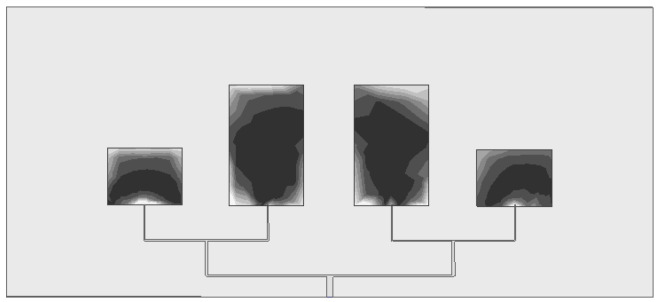
Electric field distribution of element.

**Figure 6:**
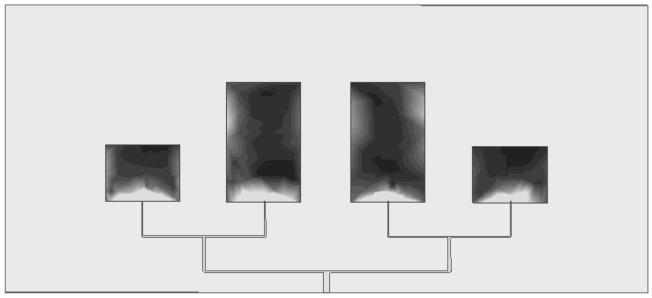
Magnetic field distribution of element.

The simulation results of the electric field and magnetic field distribution of the elements show that, as shown in Figure 5 and Figure 6, the electromagnetic field radiation area of the antenna array is highly concentrated in the central area of the elements, and the radiation direction points directly to the front of the elements without obvious sidelobe and backlobe radiation. This characteristic indicates that the antenna has extremely strong directivity, can effectively suppress the electromagnetic interference of the surrounding environment, and only receives the target fault signals in the direction of the heat dissipation hole, improving the anti-interference performance of detection from the hardware level.

#### 3.1.4. Radiation Direction Characteristics

**Figure 7:**
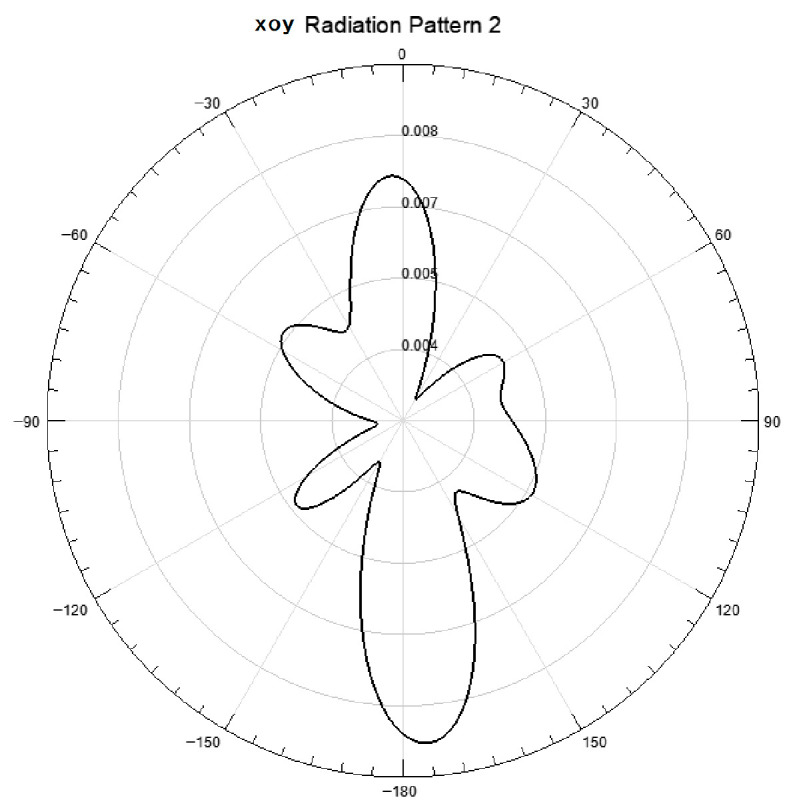
XOY diagram.

**Figure 8:**
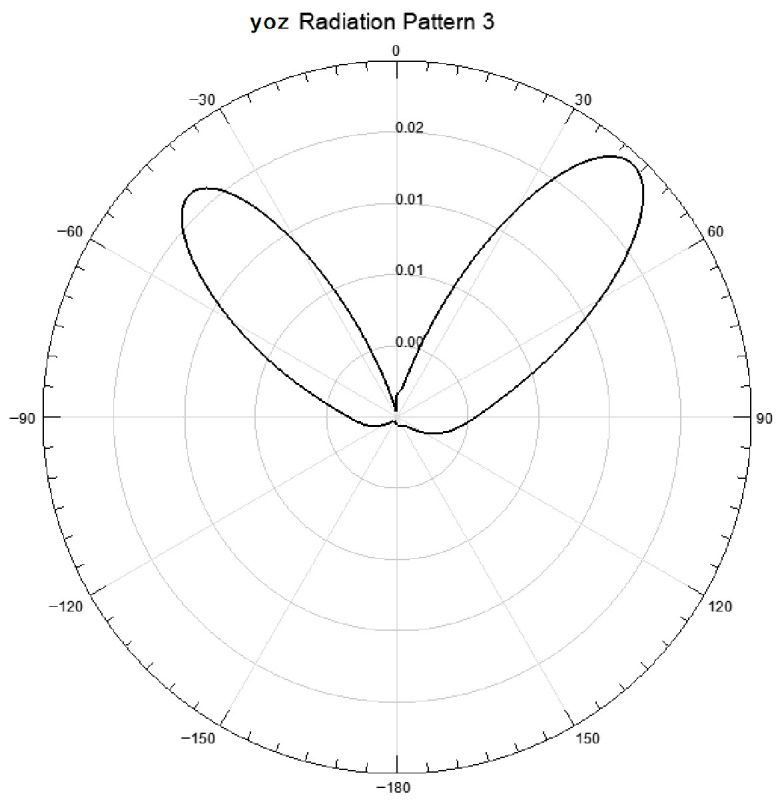
YOZ diagram.

**Figure 9:**
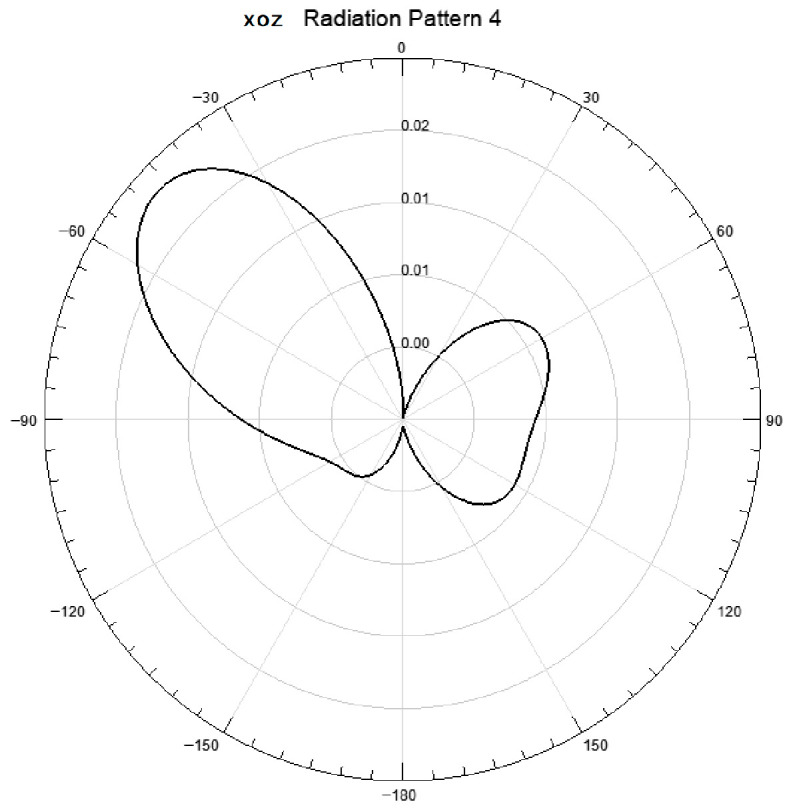
XOZ diagram.

**Figure 10:**
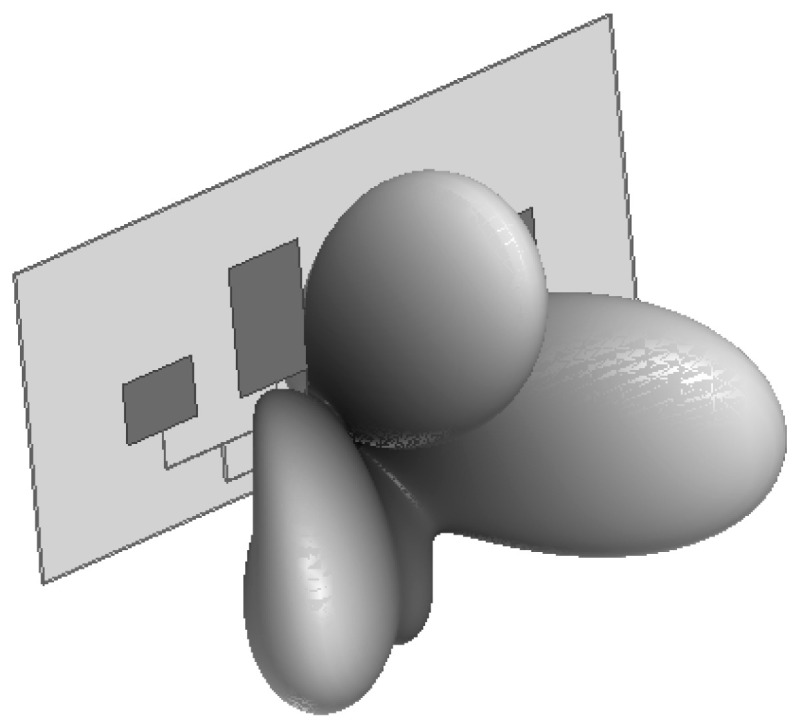
Three-dimensional beam diagram.

The two-dimensional radiation pattern (Figure 7, Figure 8 and Figure 9) and three-dimensional radiation gain pattern (Figure 10) of the antenna array show that the array forms three extremely narrow high-gain beams in the front detection area with a beam width < 30° and uniform gain distribution (5.3~5.9 dB); the three beams can fully cover the entire area of the heat dissipation hole at the tail of the induction motor without detection blind areas. The narrow beam characteristic further improves the anti-interference ability and detection sensitivity of the antenna, ensuring the accurate capture of weak discharge electromagnetic wave signals penetrating through the heat dissipation hole.

### 3.2. Simulation of Electromagnetic Wave Transmission Characteristics of Insulation Faults

**Figure 11:**
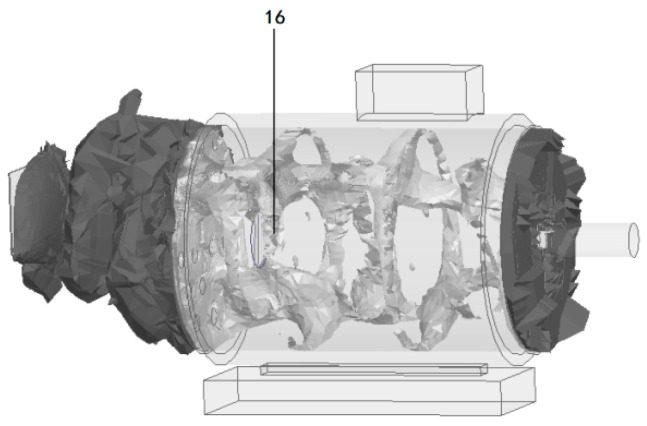
Brush position fault.

**Figure 12:**
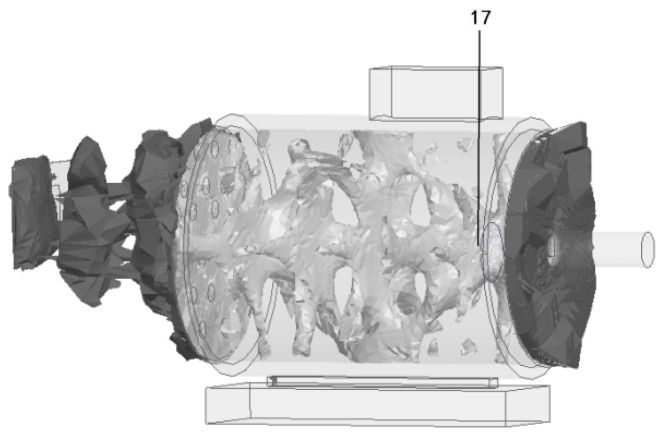
Front air gap fault.

**Figure 13:**
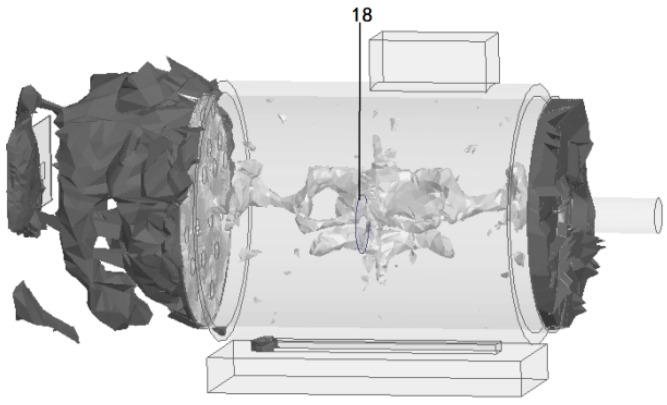
Middle air gap fault.

**Figure 14:**
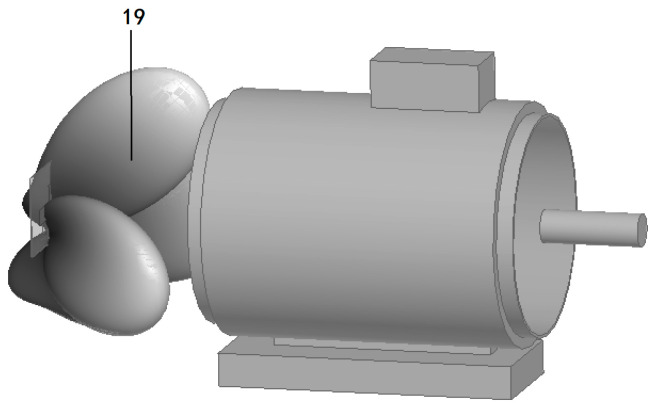
Beam coverage of antenna array.

The electromagnetic field distribution and transmission characteristics when early insulation faults occur at different positions inside the induction motor are simulated and analyzed, as shown in Figure 11, Figure 12, Figure 13 and Figure 14. The simulation results verify the following conclusions:

(1) Brush position fault: The electromagnetic waves generated by the discharge of insulation defects at the brush can effectively penetrate to the outside of the motor through the heat dissipation hole with no obvious attenuation of signal strength; only weak electromagnetic waves can penetrate through the gap of the front cover plate of the motor, and no electromagnetic waves penetrate through other metal casing areas.
(2) Front air gap fault: The electromagnetic waves generated by insulation discharge in the front air gap of the stator propagate to the tail along the internal space of the motor and radiate stably to the outside of the motor through the heat dissipation hole, which can be effectively received by the antenna array.
(3) Middle air gap fault: The insulation discharge electromagnetic waves in the middle air gap of the stator can still be transmitted to the outside of the motor through the heat dissipation hole as the only channel after multiple reflections inside the motor with no obvious signal loss.
(4) Beam coverage: The three narrow beams of the antenna array can fully cover the electromagnetic wave radiation area of the heat dissipation hole. No matter where the insulation fault occurs inside the motor, the discharge electromagnetic waves can be accurately captured without detection blind areas.

The simulation results further verify that the heat dissipation hole is the main effective channel for the outward transmission of electromagnetic waves inside the induction motor, and the designed microstrip antenna array can realize the high-sensitivity and blind area-free directional reception of electromagnetic wave signals in this channel, providing a solid simulation basis for the non-intrusive detection of early insulation faults of induction motors.

## 4. Experimental Test

### 4.1. Construction of Experimental System

**Figure 15:**
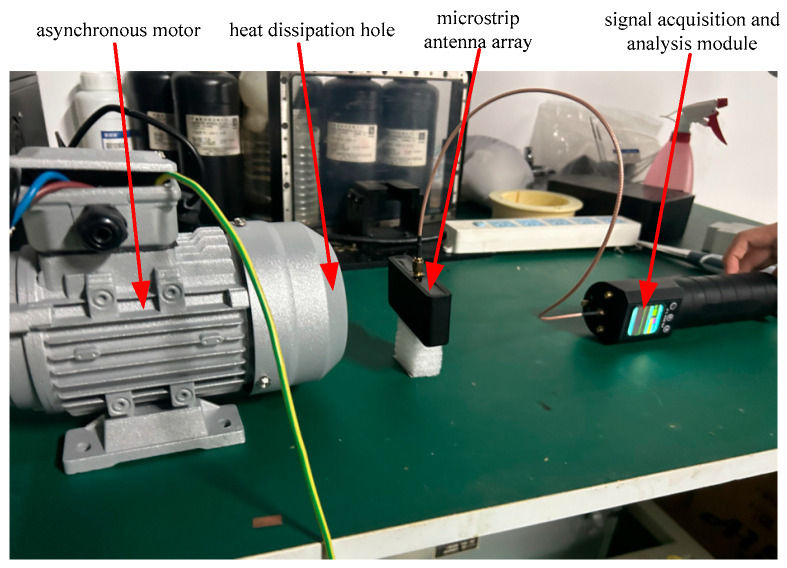
Schematic diagram of experimental system.

To verify the effectiveness, detection sensitivity, anti-interference performance, and engineering adaptability of the proposed non-intrusive detection method based on the microstrip antenna array in practical applications, a full-condition experimental system for detecting early insulation faults of induction motors is built in line with the actual working conditions of industrial sites. The system consists of four parts: a test motor platform (with built-in fault defects), a microstrip antenna array, a signal acquisition and analysis module (the sampling rate is 10 MHz, the dynamic range is 60 dB, and the system noise figure is less than 1.5 dB), and an environmental interference module, as shown in Figure 15. The hardware parameters, functions, and selection basis of each module are strictly in line with the actual industrial site, ensuring the authenticity and engineering transferability of the experimental results.

The microstrip antenna array and the signal acquisition and analysis module both adopt the non-intrusive non-contact installation method: the antenna array is installed 8 cm directly behind the heat dissipation hole at the tail of the motor through a fixed bracket, and the antenna beam fully points to the heat dissipation hole area without any mechanical contact with the motor, without affecting the normal operation of the motor, and fully meeting the actual requirements of online monitoring of operating equipment in industrial sites.

#### 4.1.1. Test Motor Platform

The industrial general three-phase induction motor (Zhongxiang Xinyu Electromechanical Manufacturing Co., Ltd., Zhongxiang, Hubei, China, Model: YE802-4) is selected as the core test object, whose parameters are consistent with the mainstream operating motors in industrial sites: rated power 0.75 kW, rated voltage 380 V, rated current 3.37 A, rated speed 1400 r/min, diameter of the tail heat dissipation hole 18 cm, F-class insulation for the stator winding, and cast aluminum squirrel-cage structure for the rotor.

To realize the precise control of motor working conditions, a motor control sub-platform is built with supporting facilities, including a frequency converter, an AC contactor, a thermal relay, and a three-phase voltage regulator, which can realize stepless speed regulation of 0~1400 r/min; at the same time, a speed sensor and a current and voltage acquisition module are equipped to monitor the motor operation state parameters in real time, ensuring the stable and controllable motor working conditions during the experiment.

#### 4.1.2. Fault Simulation Module

**Figure 16:**
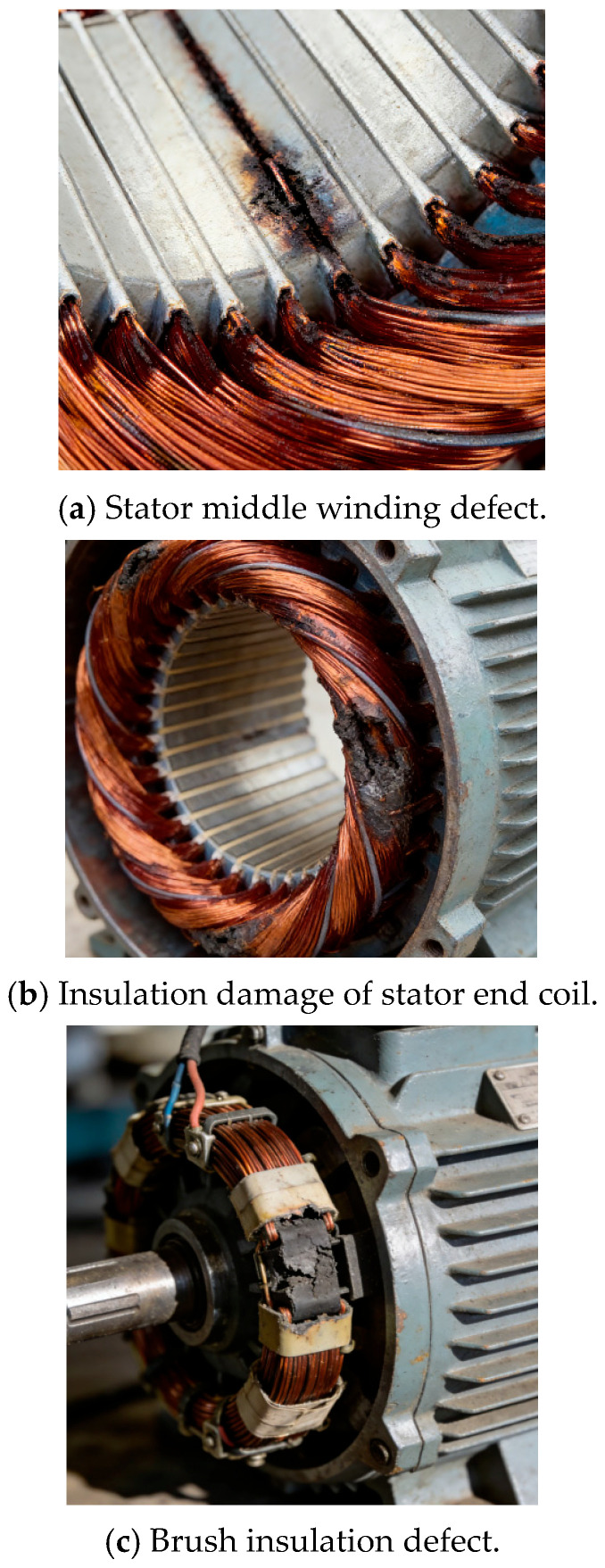
Actual defect shape.

Based on the actual occurrence law and evolution process of insulation faults of industrial induction motors, the artificial fault-making method is adopted to simulate different degrees of early insulation faults at key positions with high incidence of insulation faults such as the stator end coil, stator middle winding, brush and collector ring contact, and stator air gap. The fault-making method is in line with the formation process of actual faults. The specific fault simulation scheme is shown in Table 6, and the actual defect shape is shown in Figure 16.

#### 4.1.3. Environmental Interference Simulation Module

To simulate the complex electromagnetic interference environment of industrial sites, an interference simulation sub-platform is built, including a base station signal simulator, an industrial electromagnetic interference generator, and a white noise generator, which can realize the simulation of different types and intensities of interference signals:

(1) Base station signal simulator: This simulates the strong interference signals of mobile base stations at 900 MHz, 1.8 GHz, and 2.4 GHz;
(2) Industrial electromagnetic interference generator: This simulates the electromagnetic noise (frequency ≤ 300 MHz) generated by industrial equipment such as frequency converters and AC contactors;
(3) White noise generator: This simulates the random environmental white noise.

This module can flexibly adjust the amplitude and frequency of interference signals to realize the environmental simulation of different interference intensities and types, which is used to comprehensively test the anti-interference performance of the proposed detection method.

### 4.2. Experimental Test Scheme

This experiment is carried out in four stages, successively completing the single-condition early fault detection, multi-condition composite fault detection, and complex environment anti-interference test. Each stage is repeated 100 times. Statistical methods are used to analyze the experimental data, calculate the average value, standard deviation, and qualification rate, ensuring the reliability and repeatability of the experimental results.

Basic experimental settings: The basic motor working condition is rated load (0.75 kW) and rated speed (1400 r/min). The test starts after 30 min of operation to reach the thermally stable state. Each signal acquisition lasts for 60 s, and the signal characteristic parameters (discharge pulse amplitude, discharge pulse count, signal energy) at the detection frequencies of 1.14 GHz and 2.23 GHz are extracted as the basis for fault discrimination.

### 4.3. Heat Dissipation Hole Penetration Loss Experiment

To verify the difference in electromagnetic wave transmission characteristics between the metal motor casing and the rear heat dissipation hole, and to obtain quantitative penetration loss data, a dedicated test platform is established. Furthermore, a comparative experiment among a single-element antenna, the proposed array antenna, and a conventional commercial UHF PD sensor is supplemented to fully demonstrate the detection superiority of the array scheme, providing a solid physical basis for non-intrusive PD detection of motors.

#### 4.3.1. Experimental Setup and Test Scheme

The experimental system consists of a signal source, a power amplifier, a transmitting antenna, receiving devices (a single-element antenna, the proposed four-element array antenna, and a conventional UHF PD sensor), a spectrum analyzer, a tested motor, and fixed brackets.

(1) Transmitting antenna: This is placed inside the motor near the winding area to simulate the electromagnetic radiation source of partial discharge.
(2) Receiving devices: The single-element antenna, the four-element array antenna, and the conventional UHF PD sensor are sequentially installed at the outer center of the rear heat dissipation hole. All receivers keep the same placement distance, height, and orientation to ensure a single-variable test condition.
(3) Test frequency band: The frequency is set at 300 MHz–3 GHz, with focused scanning performed at 1.14 GHz and 2.23 GHz.

Test steps:

(1) Calibrate the free-space link loss as the reference baseline.
(2) Place the transmitting antenna inside the motor and close the end cover. Measure the received power under full metal shielding using the three receivers separately.
(3) Maintain the internal transmitting position unchanged and test the received power when electromagnetic waves radiate outward only through the rear heat dissipation hole.
(4) Calculate the penetration loss under different propagation paths, and repeat each test 10 times to obtain averaged data.
(5) Compare the signal amplitude, signal-to-noise ratio, angular adaptability, and sensitivity among the three detection devices.

#### 4.3.2. Experimental Results and Analysis

(1) Penetration loss of the metal motor casing

When electromagnetic waves penetrate the closed metal casing, the received signal is almost completely attenuated, with an average penetration loss greater than 82 dB. The result indicates that internal discharge electromagnetic waves cannot effectively radiate outward through the metal shell.

(2) Penetration loss of the rear heat dissipation hole

The heat dissipation hole presents low signal attenuation. The penetration losses at 1.14 GHz and 2.23 GHz are 6.8 dB and 8.3 dB, respectively, both lower than 10 dB. The honeycomb heat dissipation hole serves as the dominant transmission channel for internal UHF PD signals.

(3) Performance comparison among the single-element antenna, array antenna, and conventional UHF sensor

Under identical experimental conditions, the conventional UHF PD sensor exhibits limited gain, low signal amplitude, and poor angular immunity. The single-element antenna improves performance slightly but still lacks gain and directivity. In contrast, the proposed four-element array antenna realizes obvious beam synthesis and directional gain enhancement. It achieves the highest received signal amplitude, optimal signal-to-noise ratio, and strongest anti-interference capability. The array structure effectively reduces detection errors caused by angular offset and weak discharge signals.

In conclusion, the metal motor casing has excellent shielding performance for UHF electromagnetic waves, while the rear heat dissipation hole is the only reliable outward radiation path for internal discharge signals. Compared with the single-element antenna and the conventional UHF PD sensor, the proposed array antenna possesses superior sensitivity, angular adaptability, and anti-interference ability. This study provides credible experimental support for the non-intrusive PD detection method based on an external antenna array aiming at motor heat dissipation holes.

### 4.4. Single-Condition Early Insulation Fault Detection Experiment

#### 4.4.1. Experimental Process

**Figure 17:**
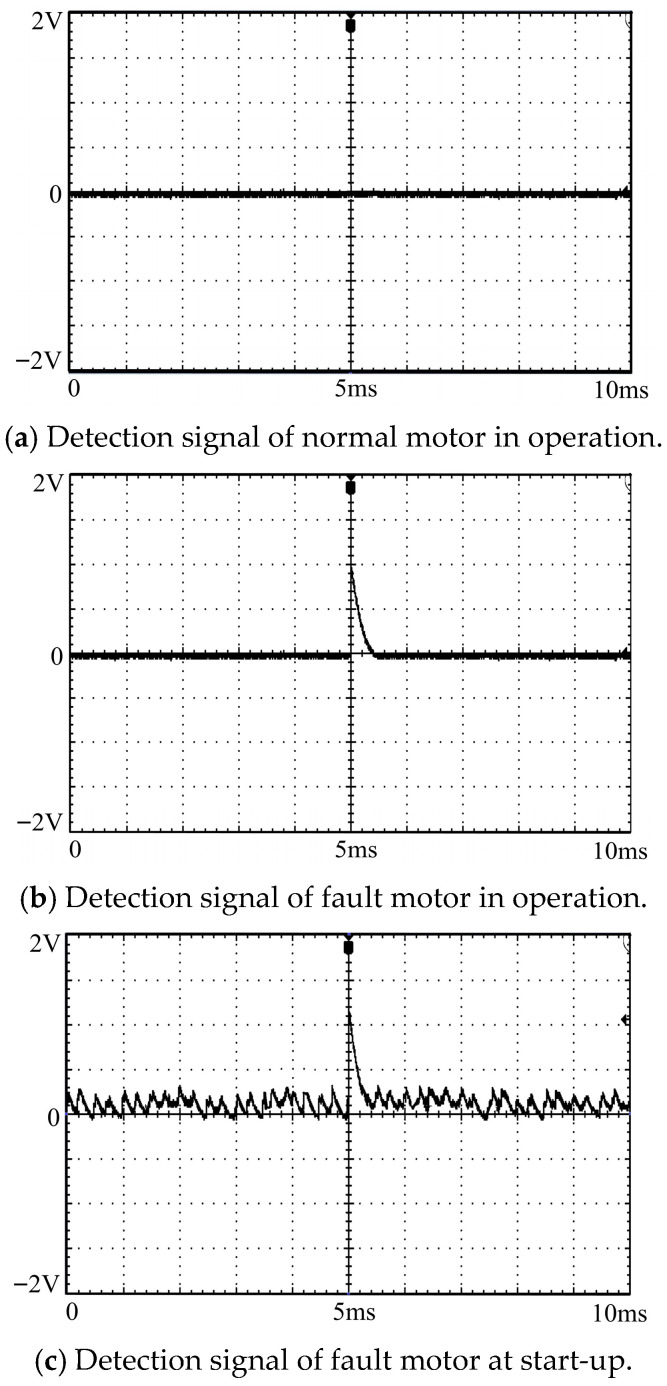
Detection signals of normal motor and fault motor in operation.

Under the single motor working condition (rated load, rated speed), the comparative detection is carried out on four types of early insulation fault motors (stator middle winding ablation, stator end coil insulation damage, brush insulation defect, stator air gap insulation breakdown) and the normal motor respectively. The experimental steps are as follows:

(1) Start the normal motor and the fault motor respectively, adjust to the rated load and rated speed, run for 30 min to reach the thermal stable state, and record the motor operation state parameters (current, voltage, speed);
(2) Turn on the microstrip antenna array detection module and the signal acquisition and analysis module, collect the electromagnetic wave signal for 60 s, and complete the signal preprocessing (filtering, denoising, amplification);
(3) Extract the characteristic parameters such as discharge pulse amplitude and discharge pulse count of the partial discharge signal at the frequencies of 1.14 GHz and 2.23 GHz;
(4) Compare the signal characteristic differences between the fault motor and the normal motor, as shown in Figure 17, determine the fault discrimination threshold, and complete the fault identification.

Repeat the above steps 100 times and record the fault identification results and signal characteristic parameters of each detection.

#### 4.4.2. Experimental Results and Analysis

The signal characteristic analysis results show that the normally operating induction motor will not generate electromagnetic wave signals at the detection frequencies of 1.14 GHz and 2.23 GHz, so there are no obvious discharge signal characteristics; while the fault motor has obvious discharge signal peaks at the above two detection frequencies, and the signal characteristics of different fault types are differentiated:

(1) The discharge signal of the stator middle winding ablation fault has a large amplitude and stable pulse count, because the discharge electromagnetic waves generated by this fault can still be stably radiated to the outside of the motor through the heat dissipation hole as the only channel after multiple reflections inside the motor, and the signal frequency band is highly matched with the detection frequency of the antenna array with no obvious signal loss;
(2) When the motor starts and the speed is suddenly increased, the electromagnetic wave background interference generated by the motor is relatively strong, but the fault discharge signal is still significantly higher than the background noise, as shown in Figure 17c, and the discharge signal can be accurately judged through the preset threshold.

Statistical methods are used to analyze the 100 repeated tests’ data, and the results are shown in Table 7. The recognition rate of the proposed method for the four types of early insulation faults all reaches 97%; among them, for the stator middle winding ablation defect, the average signal-to-noise ratio of the detection signal reaches 38.5 dB, which is far better than other fault types, indicating that the method has extremely high detection sensitivity and accuracy for typical early insulation faults such as stator middle winding ablation.

### 4.5. Multi-Condition Composite Fault Detection Experiment

#### 4.5.1. Experimental Design

In industrial sites, insulation faults of induction motors often do not occur alone but in the form of superposition of multiple faults. To verify the detection ability of the proposed method for the multi-fault superposition scenario, typical composite insulation fault types in industrial sites are simulated, and two or more types of early insulation faults are simultaneously made on the test motor. The specific composite fault combination scheme is shown in Table 8. The motor working condition is kept at a rated load and a rated speed.

After the motor reaches thermal stability, the electromagnetic wave signal is collected for 60 s, and the amplitude characteristics, pulse frequency characteristics, and signal energy characteristics of the discharge signal at the detection frequency are extracted. The fault type and fault superposition situation are discriminated by the multi-characteristic fusion algorithm; each composite fault type is tested 100 times repeatedly, and the fault recognition rate and characteristic extraction accuracy are counted.

#### 4.5.2. Experimental Results and Analysis

The statistical results of the composite fault detection experiment are shown in Table 9. The recognition rate of the proposed method for binary composite faults all reaches 97%, and the recognition rate for ternary composite faults is 93%.

From the perspective of signal characteristics, the discharge signal of the composite fault is the superposition characteristic of each single fault signal without generating new frequency components, and the discharge pulse characteristics of each fault can be effectively separated by the signal decoupling algorithm. For example, in the “end insulation damage + brush insulation defect” composite fault, the discharge pulse frequency of the brush fault is higher (about 500 Hz), and the discharge pulse amplitude of the end fault is larger. The two fault types can be accurately distinguished through the dual-characteristic analysis of pulse frequency and amplitude.

The signal-to-noise ratio of the ternary composite fault is slightly lower (31.6 dB) due to the superposition of multi-fault discharge signals and the cancellation of some pulse signals, but the overall signal is still significantly higher than the background noise of the normal motor, which proves that the method still has reliable detection ability in the composite fault scenario.

### 4.6. Complex Environment Anti-Interference Test Experiment

#### 4.6.1. Experimental Design

There are multiple electromagnetic interferences such as strong mobile base station signals, industrial electromagnetic interference, and environmental white noise in industrial sites. To verify the anti-interference performance of the proposed detection method, based on the built environmental interference simulation module, the interference environment with different intensities and types in industrial sites is simulated, and 3 interference levels (low, medium, high) are set. The specific interference parameter settings are shown in Table 10.

Taking the stator middle winding ablation fault motor as the test object, the electromagnetic wave signals at the detection frequencies are collected under different interference levels, the anti-interference margin (the ratio of fault signal amplitude to interference signal amplitude) is calculated, and the fault recognition rate is counted to verify the adaptability of the method in the complex interference environment.

#### 4.6.2. Experimental Results and Analysis

**Figure 18:**
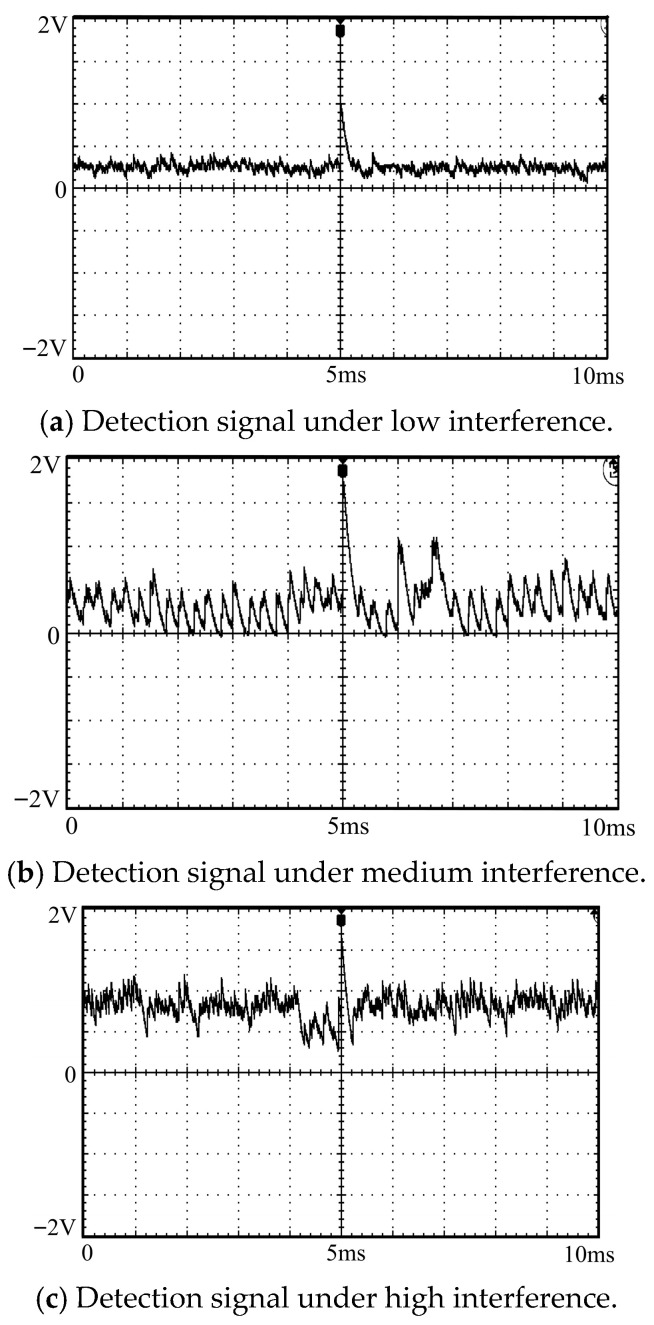
Detection signals under different interferences.

The signal characteristic analysis results show that the addition of low and medium interference will slightly reduce the signal-to-noise ratio of the detection signal, as shown in Figure 18a,b, but basically does not affect the extraction of partial discharge pulse signals; when high interference is added, the background noise is significantly enhanced, but the partial discharge pulse signal generated by the fault is still clearly distinguishable and can be effectively extracted through the signal preprocessing algorithm, as shown in Figure 18c.

The test results of anti-interference performance in complex environments are shown in Table 11. The proposed detection method achieves a fault recognition rate of 91% under both low and medium interference levels, indicating that the interference signals have no significant impact on fault detection. Under high-interference conditions, the fault recognition rate still reaches 85%. Only a few tests show a slight decrease in signal-to-noise ratio due to the superposition of white noise and industrial electromagnetic interference, but the fault signals can still be accurately identified by the signal enhancement algorithm.

The experimental results show that the designed microstrip antenna array can effectively shield the interference signals in the non-heat dissipation hole direction due to its narrow beam and strong directivity characteristics, and the detection frequencies avoid the base station and noise frequency bands, greatly improving the anti-interference ability of the detection method from the hardware level; combined with the signal preprocessing and enhancement algorithm, the influence of interference signals is further suppressed from the software level, ensuring that the proposed method still has stable and reliable detection performance in the complex electromagnetic environment of industrial sites.

## 5. Conclusions

Aiming at the problems of high difficulty in detecting early insulation faults of industrial induction motors and obvious technical limitations of traditional methods, this paper proposes a non-intrusive detection method based on a microstrip antenna array. Through theoretical analysis, simulation design, and industrial-grade experimental verification, the following core conclusions are obtained:

(1) The early insulation discharge of induction motors will radiate electromagnetic waves in the frequency band of 300 MHz~3 GHz, and the heat dissipation hole at the tail is the main effective channel for the outward transmission of such electromagnetic waves, providing an important physical basis for non-intrusive electromagnetic wave detection; the detection frequencies are designed as 1.14 GHz and 2.23 GHz, which can effectively avoid the motor operation noise frequency band (≤300 MHz) and the strong interference frequency band of mobile base stations (900 MHz, 1.8 GHz, 2.4 GHz), ensuring the purity of fault signal extraction from the frequency domain level.
(2) The designed four-element microstrip antenna array has good output impedance matching, forms three high-gain narrow beams at the detection frequencies, and realizes full-coverage and blind area-free detection of the heat dissipation hole. Compared with the omnidirectional antenna, the receiving power is increased by four times and the equivalent detection distance is increased by two times, which can accurately extract the weak discharge electromagnetic wave signals penetrating through the heat dissipation hole.
(3) The experimental results show that the recognition rate of the method for early insulation faults under single and composite conditions reaches 94% and 90% respectively, and remains above 85% in the complex industrial electromagnetic interference environment; at the same time, the non-intrusive installation method does not require power failure and disassembly, and is fully adapted to the online monitoring needs of industrial operating equipment.
(4) This method solves the technical defects of traditional vibration, temperature, and electrical quantity analysis methods, such as being susceptible to background noise interference, low detection sensitivity, and complex installation, provides a new technical scheme for the online monitoring of the insulation state of industrial induction motors, and has good engineering application value.

## Figures and Tables

**Table 1:** Performance comparison of array antennas with different element numbers.

Number of Elements N	Array Gain/dBi	Multiple of Receiving Power Improvement	Effective Sensitivity Improvement/dB	Multiple of Equivalent Detection Distance Improvement
1	0.0	1	0.0	1.0
2	3.0	2	3.0	1.4
4	6.0	4	6.0	2.0
8	9.0	8	9.0	2.8

**Table 2:** Receiving frequencies and gains of high-frequency elements with different heights.

Height of High-Frequency Element	First Receiving Frequency and Gain	Second Receiving Frequency and Gain	Third Receiving Frequency and Gain
36.1 mm	1.68 GHz, 4 dB	1.89 GHz, 9 dB	2.24 GHz, 12 dB
33.1 mm	1.68 GHz, 8 dB	1.99 GHz, 13 dB	2.24 GHz, 9 dB
28.1 mm	2.23 GHz, 15 dB	None	None
23.1 mm	2.21 GHz, 15 dB	2.67 GHz, 11 dB	None
20.1 mm	2.20 GHz, 11 dB	2.86 GHz, 16 dB	None

**Table 3:** Receiving frequencies and gains of low-frequency elements with different heights.

Height of Low-Frequency Element	First Receiving Frequency and Gain	Second Receiving Frequency and Gain	Third Receiving Frequency and Gain
52 mm	1.28 GHz, 7.5 dB	1.71 GHz, 4.4 dB	2.58 GHz, 7.7 dB
55 mm	1.23 GHz, 9 dB	2.45 GHz, 9.2 dB	None
60 mm	1.14 GHz, 16 dB	None	None
65 mm	1.03 GHz, 16 dB	2.04 GHz, 13 dB	None
68 mm	1.0 GHz, 18 dB	1.99 Hz, 8 dB	None

**Table 4:** Beam gains with different spacing between high-frequency and low-frequency elements.

Spacing Between High-Frequency and Low-Frequency Elements	Maximum Gain of Left Beam	Maximum Gain of Middle Beam	Maximum Gain of Right Beam
15 mm	6.1 dB	0.1 dB	6.3 dB
18 mm	5.8 dB	2.1 dB	5.1 dB
23 mm	5.3 dB	5.9 dB	5.6 dB
28 mm	4.1 dB	7.2 dB	3.5 dB
31 mm	6.7 dB	0.4 dB	6.5 dB

**Table 5:** Beam gains with different spacing between high-frequency elements.

Spacing Between High-Frequency Elements	Maximum Gain of Left Beam	Maximum Gain of Middle Beam	Maximum Gain of Right Beam
36 mm	2.3 dB	6.4 dB	2.6 dB
31 mm	3.3 dB	7.4 dB	3.6 dB
23 mm	5.3 dB	5.9 dB	5.6 dB
15 mm	4.4 dB	6.9 dB	5.1 dB
10 mm	3.1 dB	7.3 dB	4.5 dB

**Table 6:** Simulation scheme of early insulation faults of induction motors.

Fault Type	Simulation Position	Fault-Making Method
Insulation damage of stator end coil	Stator winding end	Ablate the insulating paint on the coil by high-temperature heating to simulate the insulating paint ablation caused by coil heating
Ablation of stator middle winding	Between the turns of stator middle winding	Ablate the anti-corona layer of the stator bar by high-temperature heating to simulate the anti-corona layer ablation caused by bar heating
Brush insulation defect	Brush and collector ring contact	Polish and ablate the brush surface by high-temperature heating to form a conductive gap
Insulation breakdown of stator air gap	Between stator and rotor air gap	Place conductive impurities on the stator and rotor to simulate intermittent spark discharge

**Table 7:** Experimental results of single-condition early insulation fault detection.

Fault Type	Fault Recognition Rate	Average Signal-To-Noise Ratio (dB)
Ablation of stator middle winding	97%	37.5
Insulation damage of stator end coil	94%	34.1
Brush insulation defect	96%	35.7
Insulation breakdown of stator air gap	95%	34.5

**Table 8:** Simulation scheme of composite early insulation faults.

Composite Fault Number	Fault Combination Type	Simulation Position and Fault-Making Method
1	Stator end insulation damage + brush insulation defect	Ablate the insulating paint on the stator winding end + polish the brush surface to form a conductive gap
2	Stator middle winding ablation + air gap insulation breakdown	Ablate the anti-corona layer of the stator middle bar + place conductive impurities in the stator–rotor air gap
3	End insulation damage + middle winding ablation + air gap breakdown	Ablate the winding end + ablate the anti-corona layer of the middle bar + place conductive impurities in the air gap

**Table 9:** Experimental results of composite early insulation fault detection.

Composite Fault Number	Fault Combination Type	Fault Recognition Rate	Average Signal-To-Noise Ratio (dB)
1	End insulation damage + brush insulation defect	97%	35.2
2	Middle winding ablation + air gap insulation breakdown	90%	31.6
3	End + middle + air gap composite fault	94%	33.8

**Table 10:** Simulation parameters of electromagnetic interference in industrial sites.

Interference Level	Interference Type	Interference Parameter Setting
Low interference	Base station signal + weak industrial electromagnetic interference	900 MHz/1.8 GHz base station signal (power 10 mW); industrial electromagnetic interference (frequency ≤ 300 MHz, power 5 mW)
Medium interference	Base station signal + medium-intensity industrial electromagnetic interference + white noise	900 MHz/1.8 GHz/2.4 GHz base station signal (power 20 mW); industrial electromagnetic interference (power 10 mW); white noise (signal-to-noise ratio 20 dB)
High interference	Strong base station signal + high-intensity industrial electromagnetic interference + white noise	900 MHz/1.8 GHz/2.4 GHz base station signal (power 50 mW); industrial electromagnetic interference (power 20 mW); white noise (signal-to-noise ratio 10 dB)

**Table 11:** Experimental results of complex environment anti-interference test.

Interference Level	Fault Recognition Rate	Average Signal-To-Noise Ratio (dB)
Low interference	97%	35.2
Medium interference	91%	32.5
High interference	85%	25.3

## Data Availability

The original contributions presented in this study are included in the article. Further inquiries can be directed to the corresponding author.
